# A decade of data from a specialist statewide child and adolescent eating disorder service: does local service access correspond with the severity of medical and eating disorder symptoms at presentation?

**DOI:** 10.1186/s40337-014-0032-0

**Published:** 2014-10-30

**Authors:** Jeremy Alman, Kimberley J Hoiles, Hunna J Watson, Sarah J Egan, Matthew Hamilton, Julie McCormack, Julie Potts, David A Forbes, Chloe Shu

**Affiliations:** Eating Disorders Program, Child and Adolescent Mental Health Service, Child and Adolescent Health Service, Roberts Road, Subiaco, Western Australia Australia; School of Psychology and Speech Pathology, Division of Health Sciences, Curtin University, Kent St, Bentley, Western Australia Australia; School of Paediatrics and Child Health, Faculty of Medicine, Dentistry and Health Sciences, The University of Western Australia, 35 Stirling Highway, Crawley, Western Australia Australia; UNC Center of Excellence for Eating Disorders, Department of Psychiatry, Neurosciences Hospital, 101 Manning Drive, Chapel Hill, North Carolina USA; Princess Margaret Hospital for Children Eating Disorders Program, GPO Box D184, Perth, 6840 Western Australia Australia

**Keywords:** Adolescent, Child, Eating disorders, HOPE Project, Medical complications, Rural

## Abstract

**Background:**

Eating disorders affect up to 3% of children and adolescents, with recovery often requiring specialist treatment. A substantial literature has accrued suggesting that lower access to health care services, experienced by rural populations, has a staggering effect on health-related morbidity and mortality. The aim of this study was to evaluate whether lower service access foreshadowed a more severe medical and symptom presentation among children and adolescents presenting to a specialist eating disorders program.

**Method:**

The data source was the Helping to Outline Paediatric Eating Disorders (HOPE) Project registry (*N* ~1000), a prospective ongoing registry study comprising consecutive paediatric tertiary eating disorder referrals. The sample consisted of 399 children and adolescents aged 8 to 16 years (*M* =14.49, 92% female) meeting criteria for a DSM-5 eating disorder.

**Results:**

Consistent with the hypotheses, lower service access was associated with a lower body mass index *z*-score and a higher likelihood of medical complications at intake assessment. Contrary to our hypothesis, eating pathology assessed at intake was associated with higher service access. No relationship was observed between service access and duration of illness or percentage of body weight lost.

**Conclusions:**

Lower service access is associated with more severe malnutrition and medical complications at referral to a specialist eating disorder program. These findings have implications for service planning and provision for rural communities to equalize health outcomes.

## Background

Eating disorders, including anorexia nervosa (AN), bulimia nervosa (BN), and other types of eating disorders are estimated to affect between 0.5% to 3% of children and adolescents [1-3]. There are serious consequences of eating disorders in this age group, including risks to growth and brain development and life-threatening medical complications [1,4]. Mortality rates of individuals with eating disorders are amongst the highest of all psychiatric disorders, particularly for AN, due to medical complications and suicide [5-8]. Eating disorders may require intensive treatment programs to facilitate recovery [9]. This is a concern in the context of rural areas where physical, cultural, and systemic barriers to accessing health services may contribute to increased medical compromise [10]. Very little research has investigated impact of service access or differences between rural and metropolitan populations with eating disorders in their clinical and medical presentation [11,12].

The nature of rural–urban health differential remains unclear with factors such as socioeconomic status, ethnicity, geographical location and access to health services likely contenders to health disadvantage in rural areas, when it is observed [10]. Distance from services may make health care more difficult to access, although in smaller countries this issue is less apparent, suggesting that a critical distance is required before the disadvantage becomes measurable in health behavior or outcomes. In Australia, the standardised mortality ratio for Australian children and adolescents in inner regional areas is 1.26, increasing to 3.44 in very remote areas [13]. Service access may impact capacity for prevention, early identification, and treatment. For example lower levels of screening may occur, therefore reducing detection rates and delaying diagnosis, and treatment may be delayed or inadequate due to reduced service capacity and limits in expertise.

General practitioners and nurses are often the only health service providers available in rural areas; 90% of psychiatric staff are located in major cities, this can lead to care ratios of up to 6000:1 for mental health professionals in rural settings [14]. As such, people in rural areas seeking mental health care are often transferred to the nearest metropolitan city, suggesting specialist psychological care in rural areas is inadequate [15]. This is undesirable for the treatment of child and adolescent eating disorders, as relocation away from home can be a stressor, and treatment is typically long-term and ideally involves the entire family and collaboration of a primary care physician, nutritionist, and therapist [9]. Obtaining appropriate treatment for paediatric eating disorders can be difficult without relocating to a metropolitan city or without an effectively resourced “hub-and-spoke” model. In the hub-and-spoke model, a specialist tertiary service (“hub”) provides training, education, and consultation and liaison services to health professionals operating in rural settings (“spoke”) [16].

Until present, there has been little research on service access in the context of children and adolescents with eating disorders, with only a few studies exploring differences in rural versus metropolitan areas. Van Son and colleagues [12] investigated incidence rates of eating disorders in children, adolescents, and adults in the Netherlands across three categories of urbanisation: rural, urbanised and large cities. BN incidence was 2.5 times higher in urban areas and 5 times higher in large cities compared to rural areas, with no differences in AN. In a Japanese study, clinical presentations of AN occurred primarily in patients from urban areas [17]. In an Italian adolescent sample, contrasting results were found [11]; more people with symptoms lived in rural areas when using dimensional criteria, but this trend was reversed when using categorical criteria, with more people in urban areas scoring above clinical cut-offs. An Italian study compared urban and suburban populations, and found a higher incidence of all eating disorder diagnoses with urbanisation [18]. Neither of these studies focused on service access, therefore little is known about how service access relates to clinical presentation when people with eating disorders present for specialist care, particularly whether low services access is related to higher morbidity.

### The present study

The aim of this study is to explore the association between service access and clinical presentation in treatment-seeking children and adolescents with eating disorders. Scientific literature indicating that people from rural communities often have poorer general physical and mental health outcomes than their metropolitan counterparts informs our predictions, as evidence pertaining specifically to eating disorders is lacking. We predict that children and adolescents with eating disorders who present for specialist care will have poorer physical health and more severe eating pathology as service access decreases. Specifically, we predict they will show higher duration of illness, loss of body weight, eating pathology, and medical complications, and lower body mass index (BMI) *z*-score.

## Methods

### Participants and procedure

The study is part of a larger study called the Helping to Outline Paediatric Eating Disorders (HOPE) Project, an ongoing prospective clinical cohort registry study, with 1024 participants at the time of the present study. The participation rate in the HOPE Project is approximately 97% [19]. Ethical approval for this study was obtained from the Princess Margaret Hospital for Children and Curtin University Human Research Ethics Committees.

Western Australia covers an area of 2 532 400 km^2^, which is approximately 10 times the size of the United Kingdom. The state consists of 2 430 300 people, of which 621 800 are children or adolescents [20]. Approximately 262 742 (11%) of Western Australians live in outer regional or remote areas, that is, areas that are from 3 hours to 3 days drive from the major city Perth which has the only public specialist paediatric eating disorders service in the state. The HOPE Project registry consists of children and adolescents that consecutively presented to this specialist service, Child and Adolescent Health Service Eating Disorder Program (CAHS EDP) (formerly Princess Margaret Hospital for Children Eating Disorders Program) between 1996 and 2013. All children and adolescents and their families presenting to CAHS EDP undertake routine intake assessment spanning two half-days to determine whether they have an eating disorder diagnosis according to the fifth edition of the Diagnostic and Statistical Manual (DSM-5) [21] and to evaluate mental and physical health status.

Participants were included in the study if they met DSM-5 criteria for an eating disorder and were referred from 2001, so a service access score could be assigned. DSM-5 eating disorder diagnosis was assigned retrospectively based on prospectively collected child and parent eating disorder interviews adapted from Fairburn and Coopers Eating Disorder Examination (EDE) [22] and medical records [19], and prospectively after publication of DSM-5. Participants who attended boarding school in Perth but whose family residence was outside Perth, causing ambiguity to their status, were excluded (*n* =7) as well as participants who had a postal code for which an access score could not be assigned (*n* =2). The final sample comprised 399 participants aged 8 to 16 years (*M* =14.49, 92% female).

### Measures

*The Access/Remoteness Index for Australia Plus (ARIA+)* measured service access and is a relatively stable measure of remoteness and access to services developed by the National Key Centre for Social Applications of Geographic Information Systems for the Department of Health and Aged Care [23]. The ARIA + is a continuous variable (between 0–15) with higher scores reflecting poorer service access - the following categories have been offered to describe accessibility; highly accessible (0–0.19), accessible (0.2 - 2.39) moderately accessible (2.4 - 5.92), remote (5.93 – 10.53), and very remote ([10.54 – 15] [23].

The *Socio-Economic Indexes for Areas (SEIFA)* Index of Economic Resources is a continuous measure of relative socio-economic advantage and disadvantage or a region; with higher scores indicating greater access to resources [24,25]. The SEIFA was used as a covariate as per previous research to adjust for socioeconomic differences across postcodes [13,25].

*Duration of illness (months)* was the self-reported length of time the patient had been experiencing eating disorder symptoms prior to assessment and was collected in the medical review with the family and child in attendance. *BMI z-score* assessed nutritional status and was calculated with Epi Info 7 [26], entering the patient’s height and weight collected at the medical initial assessment, sex, and age into the program. *Z*-scores are age- and sex-adjusted based on the Centres for Disease Control and Prevention [27] growth charts. *Eating pathology* was assessed with the adapted child version of the EDE [22]. The EDE has four subscales – restraint, eating concern, shape concern, and weight concern – that aggregate to a global score. Internal reliability is acceptable and all but three items have been found to have inter-rater reliability coefficients > .9 [28]. *Percentage of body weight lost* was calculated by subtracting the participant’s weight at the initial intake assessment from the maximum ever self-reported weight, dividing by maximum ever weight, then converting to a percentage. This is an imperfect method, since (unmeasured) linear growth occurs over the average 8 months illness duration, but is a sufficient proxy. *Potentially life-threatening medical complications* was a categorical variable defined by the presence of at least one medical indicator (1 = yes, 0 = no), specifically bradycardia, hypothermia, hypotension, and poor peripheral perfusion. The medical indicators were defined based on age-appropriate cut-offs [4].

### Statistical analysis

General linear and generalized linear modelling (GLM) approaches were used. The predictor for all models was ARIA + and analyses were adjusted for the confounding effects of age, sex, presence of AN, and SEIFA. Presence of AN was added as a covariate because a higher proportion of participants in lower services access presented with AN, and we wanted to be able to tease apart medical and psychological differences attributable to service access, ruling out diagnostic differences. The dependent variables of BMI *z*-score, duration of illness, percentage of body weight lost, and global EDE were modelled with GLMs. The binary dependent variables, medical complications, were modelled with a binomial distribution and a logit link function. Statistical significance was observed at the conventional alpha level of .05.

Prior to analysis, Little’s MCAR test was used to assess the pattern of missing data. All variables had less than 5% missing data, except for percentage of weight lost (15%). Missing data were assessed as missing completely at random, χ^2^ (93) = 70.44, *p* = .96, and were imputed via SPSS MVA expectation maximization.

## Results

### Sample characteristics

Although a continuous measure of service access was used in the analyses, sample characteristics (see Table 1) were categorised descriptively by accessibility classification; highly accessible, accessible, and moderately accessible, remote, and very remote. Approximately 11% of referrals were from moderate to low service access areas, which is proportional to the general population distribution. The mean age of the sample was 14.49 (*SD* = 1.54) years and the predominant diagnosis was AN (39.3%) followed by other specified feeding or eating disorder (28.8%), unspecified feeding or eating disorder (24.1%), BN (7.5%), and binge eating disorder (0.3%).

**Table 1:** **Descriptive and clinical characteristics of children/adolescents with eating disorders for all potential outcomes by accessibility classification**

	**Highly accessible (** ***N*** **= 295)**	**Accessible (** ***N*** **= 61)**	**Moderately accessible, remote and very remote (** ***N*** **= 43)**	**Total (** ***N*** **= 399)**
Age (yrs), *M* (*SD*)	14.47 (1.57)	14.70 (1.42)	14.32 (1.46)	14.49 (1.54)
Female, *n*	277 (93.8%)	52 (85.2%)	39 (90.7%)	368 (92.2%)
DSM −5 diagnosis				
Anorexia nervosa	109 (36.9%)	23 (37.7%)	25 (58.1%)	157 (39.3%)
Other specified feeding or eating disorder	94 (31.8%)	15 (24.6%)	6 (14.0%)	115 (28.8%)
Unspecified feeding or eating disorder	70 (23.6%)	15 (24.6%)	11 (25.6%)	96 (24.1%)
Bulimia nervosa	21 (7.1%)	8 (13.1%)	1 (2.3%)	30 (7.5%)
Binge eating disorder	1 (0.3%)	0	0	1 (0.3%)
Age of onset (yrs), *M* (*SD*)	13.61 (1.67)	13.72 (1.56)	13.52 (1.65)	13.62 (1.65)
Illness duration (mths), median ± IQR [range]	8.00 ± 5-12 [1, 48]	9.00 ± 6-18 [1,36]	6.00 ± 4-9 [2,78]	8.00 ± 5-12 [1,78]
BMI *z*-score, *M* (*SD*)	−1.38 (1.41)	−1.45 (1.59)	−2.29 (1.32)	−1.49 (1.45)
Percentage of body weight lost, *M (SD)*	16.35 (10.85)	17.50 (10.89)	19.82 (11.33)	16.90 (10.93)
Medical complications, *n*	101 (34.2%)	23 (37.7%)	25 (58.1%)	149 (37.3%)
Bradycardia	70 (23.7%)	14 (23.0%)	17 (39.5%)	101 (25.3%)
Hypotension	32 (10.8%)	8 (13.1%)	8 (18.6%)	48 (12.0%)
Hypothermia	12 (4.1%)	0	4 (9.3%)	16 (4.0%)
Poor peripheral perfusion	27 (9.2%)	7 (11.5%)	10 (23.3%)	44 (11.0%)
EDE-restraint	3.36 (1.73)	3.51 (1.6)	3.08 (2)	3.35 (1.74)
EDE eating concern	2.52 (1.66)	2.58 (1.68)	1.98 (1.49)	2.47 (1.65)
EDE shape concern	3.45 (1.86)	3.66 (1.72)	3.05 (1.92)	3.44 (1.85)
EDE weight concern	3 (1.86)	2.99 (1.64)	2.58 (1.75)	2.95 (1.82)
EDE global score	3.08 (1.63)	3.19 (1.43)	2.67 (1.66)	3.05 (1.60)

*Note*: Median +/− IQR [range] were reported when variable skewness (>3) was present in 1 or more group.

### Service access, medical and eating disorder characteristics at tertiary presentation

As shown in Table 1, a higher percentage of children and adolescents from lower service access areas had AN, and there was a positive association between ARIA+ scores and AN diagnosis (β = 0.12, 95% CI: 1.04 to 1.23, *p* = .01). Therefore, presence of AN was controlled for in subsequent analyses. As hypothesised, there was a significant negative association between ARIA+ and BMI *z*-score (*β =* 0.08, 95% CI: −0.13 to −0.03, *p* = .004) and medical complications (OR = 1.99, 95% CI: 1.01 to 1.21, *p* = .03), such that lower service access was related to greater medical compromise. Contrary to our predictions, ARIA+ and global EDE scores were significantly negatively associated (*β =* 0.08, 95% CI: −0.14 to −0.02, *p* = .01), such that as distance from specialist service increased, pathology was lower. There was a non-significant relationship between ARIA+ and percentage of body weight lost (*β =* 0.26, 95%CI: −1.16 to 0.68, *p* = .22) and duration of illness even when adjusting for covariates (*β* = −0.04, 95% CI: −0.40 to 0.33, *p* = .85).

## Discussion

To the authors’ knowledge, this study is the first to investigate the association between service access and clinical characteristics of children and adolescents presenting for specialist eating disorder treatment. The study discovered that children and adolescents with eating disorders assessed at a specialist treatment setting who were located in areas with lower service access presented to specialist treatment at greater physical risk, with lower BMI *z*-score and more prevalent medical complications.

An issue to consider in the present study is referral bias, since the study described children who were ultimately referred and then assessed at a specialist service and may have more severe and complex presentations than children with eating disorders in the community who do not get referred for treatment. Generalization of the study findings to youth who are not referred to treatment is not possible, without knowledge of the broader epidemiological features of children with eating disorders in Australia. Population-based research would help to determine if clinic-referred youth are sufficiently similar to non-clinic referred youth.

Amongst the assessed youth, we noted that AN was more prevalent in patients referred from rural areas, which we considered to be an artefact of referral bias rather than a true prevalence difference, given the reproducibility of prevalence estimates of AN generally. Hence we adjusted for AN in the analyses to rule this out as a possible confound explaining differences in physical and medical compromise between rural- and locally-assessed youth.

Our findings, based on over a decade of collected data, indicate that young people presenting from areas with lower access to health services had substantially higher physical and medical morbidity related to their eating disorder. The findings are of serious concern because malnutrition and medical complications of eating disorders may lead to increased mortality [5], stunted vertical growth, cardiac complications, and osteoporosis [7,8]. There may be some parallel with depression and cancer, whilst rural areas do not always have higher prevalence of these disorders, morbidity and mortality can be worse, which may reflect rates of screening, recognition and treatment capacity in more isolated and remote areas [29,30]. An important implication of this study is that young people with eating disorders need to be supported by a health system that is accessible, relevant, and timely, regardless of residential location.

Further studies are needed to understand the role of service access in morbidity, as conceptualised in this study, and how this factor works together with other plausible explanations. It may be that other aspects, such as rural cultural values of self-reliance and survival through independence, and higher stigma or concerns about confidentiality in smaller residential areas, impede access also [10]. There may be relationships between rural status and poverty, which may increase risk for extreme weight status of either underweight through lack of nutrition, or overweight through consumption of nutrient-poor, calorie dense, but more affordable foods [31-33]. Findings from the Australian Institute of Health and Welfare [34] suggest that there is an association between low socioeconomic status and childhood rates of obesity. Such data could shed light on differences in child weight status between geographical regions. Sociocultural mechanisms are known to shape eating disorder presentation and symptomatology; future research could explore whether the clinical characteristics observed in this study are a feature of eating disorders in more remote areas, for example, lower eating pathology may correspond with differences in media and Internet access and use.

In contrast to predictions, an increase in rural status was not associated with an increase in severity of eating disorder pathology, but rather the opposite was observed. Eating pathology may not be as conspicuous as physical deterioration and weight loss, and may be less of a factor associated with the decision to refer for specialist treatment, in particular to tertiary inpatient care which is a common entry point of rural patients to metropolitan services. It may also be that this finding is less related to service access and reflects the phenomenology of eating disorders in more geographically isolated settings. Sociocultural theory proposes that urbanisation is associated with greater exposure to the thin ideal and eating concern [35] and consequently those experiencing eating disorders from rural areas may have less developed weight, shape and eating cognitions and share some similarity with those observed in non-Westernised cultures where rationales for self-starvation are more variable [36,37]. Further, the higher levels of overall BMI in rural communities may both reduce and reflect the social pressure to be thin.

Contrary to our predictions, there was no relationship between an increase in rural status and duration of illness or percentage of body weight lost for tertiary assessed children. It is reassuring that duration of illness is not different by health service access, and while not statistically significant there was a trend for greater percentage of body weight lost with lower service access that may be clinically meaningful, particularly when considered in combination with the statistically significant differences in BMI z-score and general malnourishment observed in this clinical population.

Limitations of the study include the lack of information about individuals with an eating disorder not seen by health care professionals, or seen by primary care professionals in the community without referral to specialist care. As a consequence, many young people would not be included in this study and as a result the sample is biased toward treatment-seeking patients and families. BMI-z score and percent body weight loss do not take into account changes in height from the time of premorbid weight to the time of clinic assessment, so conclusions regarding these findings are limited. Socioeconomic status of regions was controlled for in the current study; however a measure of individual wealth status was not, which could explain the association between rurality and weight status in our cohort independent of eating disorder severity and course. The EDE and other measures of eating pathology have shortcomings when applied to AN, making it difficult to compare rural and metropolitan populations with this diagnosis [38,39]. The EDE was the only measure of psychological symptoms. Future studies could investigate comorbid psychological symptoms to identify if differences exist in variables other than physical health.

## Conclusions

In conclusion, our findings suggest that lower access to health services is associated with greater danger of malnutrition and medical complications by the time the young person and their family obtains specialist care. The most important implication of our study is that individuals with low access to health services, such as those residing in rural areas, may require better access to services via telehealth and e-health, transport, accommodation assistance, dedicated outreach or training, and consultation services for families required to travel long distances to access specialist services [16]. Additionally, advocacy and education may assist to reduce the possible stigma of mental illness and help seeking in rural communities. Identifying the specific factors underlying rural–urban health differentials, such as distance from services or social or cultural impediments such as stigma or fear of loss of confidentiality, is important to the development of targeted and effective rural health policies and interventions. Further research and policy are needed to focus on improving hub and spoke models, increase access to appropriate care, and reduce health inequities in rural-dwelling communities and others who reside away from specialist care. Our study is based on data from the past decade, it is up to funders, policy makers, and health service planners and providers to work creatively into the next decade to address the needs of lower access populations.

## Abbreviations

AN: Anorexia nervosa
BN: Bulimia nervosa
BMI: Body mass index
HOPE: Helping to Outline Paediatric Eating Disorders
CAHS EDP: Child and Adolescent Health Service Eating Disorders Program
DSM-5: Diagnostic and Statistical Manual Fifth Edition
EDE: Eating Disorder Examination
ARIA+: Access/Remoteness Index for Australia Plus
SEIFA: Socio-Economic Index for Areas
GLM: Generalised linear models
